# MGMT gene promoter methylation correlates with tolerance of temozolomide treatment in melanoma but not with clinical outcome

**DOI:** 10.1038/sj.bjc.6605796

**Published:** 2010-08-24

**Authors:** J C Hassel, A Sucker, L Edler, H Kurzen, I Moll, C Stresemann, K Spieth, C Mauch, K Rass, R Dummer, D Schadendorf

**Affiliations:** 1 Skin Cancer Unit, German Cancer Research Center, University Hospital Mannheim, Mannheim, Germany; 2 German Cancer Research Center, Department of Biostatistics, Heidelberg, Germany; 3 German Cancer Research Center, Department of Epigenetics, Heidelberg, Germany; 4 Department of Dermatology, Johann Wolfgang Goethe-University, Frankfurt am Main, Germany; 5 Department of Dermatology, University of Cologne, Cologne, Germany; 6 Department of Dermatology, Saarland University Hospital, Homburg, Germany; 7 Department of Dermatology, University Hospital Zurich, Zurich, Switzerland; 8 Department of Dermatology, University Hospital Essen, Hufelandstr. 55, Essen 45122, Germany; 9 Bayer-Schering, Müllerstr. 178, Berlin D-13533, Germany

**Keywords:** MGMT, gene methylation, melanoma, therapy toxicity, temozolomide

## Abstract

**Background:**

Despite limited clinical efficacy, treatment with dacarbazine or temozolomide (TMZ) remains the standard therapy for metastatic melanoma. In glioblastoma, promoter methylation of the counteracting DNA repair enzyme *O*^6^-methylguanine-DNA-methyltransferase (MGMT) correlates with survival of patients exposed to TMZ in combination with radiotherapy. For melanoma, data are limited and controversial.

**Methods:**

Biopsy samples from 122 patients with metastatic melanoma being treated with TMZ in two multicenter studies of the Dermatologic Cooperative Oncology Group were investigated for *MGMT* promoter methylation. We used the COBRA (combined bisulphite restriction analysis) technique to determine aberrant methylation of CpG islands in small amounts of genomic DNA isolated from paraffin-embedded tissue sections. To detect aberrant methylation, bisulphite-treated DNA was amplified by PCR, enzyme restricted, and visualised by gel electrophoresis.

**Results:**

Correlation with clinical data from 117 evaluable patients in a best-response evaluation indicated no statistically significant association between *MGMT* promoter methylation status and response. A methylated *MGMT* promoter was observed in 34.8% of responders and 23.4% of non-responders (*P*=0.29). In addition, no survival advantage for patients with a methylated *MGMT* promoter was detectable (*P*=0.79). Interestingly, we found a significant correlation between *MGMT* methylation and tolerance of therapy. Patients with a methylated *MGMT* promoter had more severe adverse events, requiring more TMZ dose reductions or discontinuations (*P*=0.007; OR 2.7 (95% CI: 1.32–5.7)). Analysis of *MGMT* promoter methylation comparing primaries and different metastases over the clinical course revealed no statistical difference (*P*=0.49).

**Conclusions:**

In advanced melanoma *MGMT* promoter, methylation correlates with tolerance of therapy, but not with clinical outcome.

Therapy of metastatic melanoma still lacks efficacy because, other than surgical resection of single metastases in a subgroup of patients (Meyer *et al*, 2000; Wood *et al*, 2001), no therapeutic approach significantly improves patients’ prognosis. Chemotherapy of advanced melanoma has not yet shown any survival benefit despite extensive efforts. Although polychemotherapy procedures have achieved better response, the overall survival (OS) of patients was not improved in comparison with monochemotherapy (Rass and Hassel, 2009). Therefore, dacarbazine (DTIC) is still considered as the standard chemotherapy in melanoma (Dummer *et al*, 2008). Temozolomide (TMZ) is the oral homologue of DTIC, both act through the metabolite MTIC which subsequently induces genetic alterations by alkylation. DNA repair enzymes can partly reverse these effects and *O*^6^-methylguanine-DNA-methyltransferase (MGMT) counteracts TMZ-induced DNA alkylation.

Epigenetic silencing of the *MGMT* gene by promoter methylation of CpG islands was recently associated with prolonged survival of patients with glioblastoma who received TMZ in combination with radiotherapy (Hegi *et al*, 2005). For melanoma, few studies, with limited numbers of patients, have analysed MGMT activity and most of these did not focus on *MGMT* gene methylation, but on MGMT protein expression levels and MGMT activity. Middleton *et al* (1998) measured MGMT activity in pretreatment melanoma biopsies from 33 patients and found no predictive value for response to TMZ. Ma *et al* (2003) retrospectively investigated, by immunohistochemistry, MGMT protein expression in melanoma metastases of 79 patients with metastatic melanoma receiving DTIC as single agent or as part of combination therapy, and reported an inverse correlation between MGMT expression and clinical response with borderline significance (*P*=0.05). Recently, Rietschel *et al* (2008) treated 49 melanoma patients with an extended-dosing TMZ schedule and assessed *MGMT* promoter methylation and protein expression by methylation-specific pyrosequencing of the promoter and by immunohistochemistry, respectively. These authors did not detect any correlation of *MGMT* methylation with TMZ response.

Here, to evaluate the effect of *MGMT* methylation on response, survival, and tolerance of TMZ treatment, we examined *MGMT* gene silencing by methylation of its promoter using the COBRA (combined bisulphite restriction analysis) technique in melanoma biopsies from 122 patients with metastatic melanoma who had been treated with TMZ in two large DeCOG (Dermatologic Cooperative Oncology Group) multicenter studies.

## Materials and methods

### Patients and treatment

Melanoma biopsies were collected from patients in two randomised multicenter studies, evaluating TMZ chemotherapy as first-line treatment for stage IV melanoma from June 1992 to September 2006 carried out by the DeCOG. Patients were treated by one of three different therapy regimes: TMZ (200 mg m^−2^ per day for 5 consecutive days) alone, in combination with subcutaneous interferon-*α*2b (5 MU m^−2^ three times a week) (Kaufmann *et al*, 2005), or in combination with 100 *μ*g subcutaneous pegylated interferon-*α*2b (Spieth *et al*, 2008).

Sampling of melanoma biopsies in both trial protocols was approved by the responsible ethics committee. Of the 418 patients enrolled in the two DeCOG trials, approximately one quarter (29.2%, *n*=122) were eligible, agreed to participate in this translational research project, and provided additional written informed consent. Participation in this translational research project was dependent on evaluable biopsiable tumour, for example, in the form of skin or easily reachable lymph node metastases. Archived paraffin-embedded tissue sections were used for investigation of *MGMT* promoter methylation.

### Outcome measurements

Response was evaluated using the bi-dimensional WHO criteria, as described in the underlying clinical study protocols (Kaufmann *et al*, 2005; Spieth *et al*, 2008).The response rate (RR), defined as all patients with a complete response (CR) or partial response (PR) as best response to treatment, and the disease control rate (DCR), defined as all patients with a CR, PR, or stable disease (SD) as best response to treatment, were noted. For survival analysis, OS was measured from the start of treatment to death or last observed date alive (censored observation), and progression-free survival (PFS) was measured from the start of treatment to the date of progression or death, which ever occurred first, or last the date alive (censored observation).

Adverse events (AEs) of TMZ were monitored during treatment and graded according to the Common Toxicity Criteria. Tolerance was categorised, from excellent to poor, on the basis of the need to modify treatment (defined per protocol) mainly because of haematologic toxicity, for example, leucopenia and thrombocytopenia. ‘Excellent’ was defined as no AE reported, ‘good’ as mild AE reported without any need for dose reduction, ‘satisfactory’ as any AE requiring dose reduction, ‘moderate’ as therapy interrupted, followed by dose reduction, and ‘poor’ as any AE requiring discontinuation of treatment.

### DNA extraction

To confirm that sufficient tumour tissue was available, haematoxylin and eosin-stained slides were reviewed histologically by an independent, blinded reviewer (HK). Only lesions with a tumour content of more than 75% were included. For extraction of DNA from tissue sections, we used the QIAamp DNA Mini Kit (Qiagen, Hilden, Germany) according to manufacturer's instructions. Deparaffinised 10-*μ*m sections from each sample were used for extraction.

### COBRA

**Figure 1:**
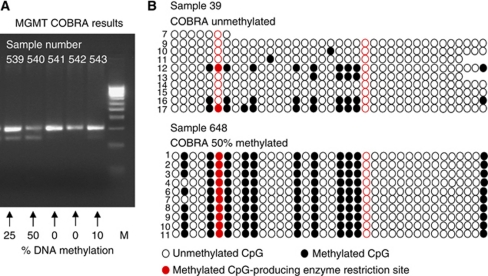
(**A**) COBRA results from five representative samples. Tumour DNA was extracted from paraffin-embedded tissue sections, bisulphite treated, amplified by PCR, *Taq*1 digested, and visualised by gel electrophoresis. For semiquantitative evaluation, percentage of cleaved and, therefore, methylated DNA (lower band) was estimated as 0, 10, 25, 50, 75, or 100% methylated (see numbers; M=marker); (**B**) Examples of bisulphite sequencing results for 10 clones of unmethylated (sample 39) and methylated (sample 648) samples in COBRA analysis: For quality control, PCR products of the COBRA analysis were extracted from the gel and cloned; each circle represents a CpG island of the MGMT promoter. The ‘unmethylated’ sample by COBRA analysis revealed, on average, 3.4 methylated CpG islands, the ‘methylated’ sample 11.8, indicative of reliable COBRA results.

Combined bisulphite restriction analysis was performed to determine aberrant methylation of CpG islands in small amounts of genomic DNA (Xiong and Laird, 1997). We introduced methylation-dependent sequence differences by sodium bisulphite treatment of genomic DNA using the EpiTect Bisulphite Kit (Qiagen) according to manufacturer's instructions. Primers used for amplifying a 295-bp PCR product of the MGMT promoter region were as follows: forward 5-TGGTAAATTAAGGTATAGAGTTTTAGG and reverse 5-AAAACCTAAAAAAAACAAAAAAAC. The PCR reactions were performed in a volume of 20 *μ*l containing 1x Ready mix (Abgene, Cambridge, UK), 2 *μ*l bisulphite-treated genomic DNA, 0.2 *μ*M of each primer, 1 mM dNTPs (Stratagene, Amsterdam, NL, USA), and 1 U Thermoprime Plus DNA polymerase (Abgene). We performed 35 cycles of amplification at 95°C for 30 s, 55°C for 30 s, and 72°C for 45 s followed by a final extension at 72°C for 3 min. PCR products were digested with restriction enzyme *Taq*I (New England Biolabs, Ipswich, MA, USA) and separated on agarose gels. For semiquantitative evaluation, percentage of cleaved and, therefore, methylated DNA of the extracted amount of genomic DNA was estimated as 0, 10, 25, 50, 75, or 100% methylated (Figure 1a).

To ensure reliable results, several analyses from different blocks of a tumour were examined, altogether 543 samples. Of these, 61 (11.2%) could not be evaluated by COBRA analysis and were considered as invalid in the analysis. The frequency of invalid samples and the methylation status of the samples were not dependent on the study centre where the biopsy was taken (*P*=0.11).

### COBRA assay validation by sequencing

Five samples defined by COBRA as methylated and five samples defined by COBRA as unmethylated were analysed by bisulphite sequencing of 33 CpG islands for quality control. PCR products were extracted from the gel and cloned using the TOPO TA cloning kit (Invitrogen, Karlsruhe, Germany) according to manufacturer's instructions.

A degree of methylation ⩾50% was observed in four of the five samples classified as ‘methylated’ in COBRA analysis, and 9.9–11.8 methylated CpGs were observed in bisulphite sequencing. Only 10% methylation with 3.8 methylated CpGs was observed in the fifth sample. For the five samples classified as unmethylated by COBRA analysis, methylation was from 3.1 to 7.7 methylated CpGs of the 33 CpGs analysed (Figure 1b, Supplementary Table 1). Together, these results confirmed that the COBRA assay adequately represents the genomic level of methylation of the *MGMT* promoter.

### Immunohistochemistry

Immunohistochemistry was conducted on 4-*μ*m sections of formalin-fixed paraffin-embedded tissue. Sections were placed on Superfrost Plus slides (Menzel, Braunschweig, Germany) and baked for 1 h at 60°C. They were then deparaffinised, hydrated, and placed in prewarmed citrate buffer and boiled in a microwave oven for 20 min. Still in the citrate buffer the slides were cooled at room temperature for 20 min. After incubation in blocking solution (Zytomed ZytoChem-Plus AP Polymer Kit, Berlin, Germany), 1 : 50 dilution of anti-MGMT clone MT23.2 (Zymed Laboratories, Amsterdam, NL, USA) was added and the slides were incubated overnight at 4°C. Slides were washed in PBS and antibody binding was visualised with Zytomed ZytoChem-Plus AP Polymer Kit and Zytomed Permanent AP Red Kit, according to manufacturer's instructions. Substrate was incubated for 10 min and slides were counterstained with Mayers Hämalaun for 30 s and mounted with Eukitt. As simultaneous negative control we used sections stained without the first antibody. As positive control colon carcinoma sections were used.

### Statistical analysis

Patient characteristics of the study population (*n*=122) were analysed using descriptive statistical methods, that is, frequency tables, median, and range. For each patient, the COBRA result from the tumour sample with the highest tumour content in the paraffin block was used. The methylation status of the first evaluable lesion (primary tumour or metastasis) of a patient was examined for its effects on the rate of RR and DCR using frequency tables (contingency tables). Instead of using the six subgroups ‘0, 10, 25, 50, 75, and 100% methylation’ as evaluated by COBRA, the subgroups were split into two groups, ‘methylated’ and ‘unmethylated’, because the number of patients did not allow analysis of so many subgroups. Fisher's exact test was used to test for differences between the methylated and non-methylated groups. Overall survival and PFS were analysed by the Kaplan–Meier method, and the two methylation groups were compared using the two-sided log-rank test. The prognostic effect of *MGMT* promoter methylation and clinical factors was investigated by use of the multivariable proportional hazard regression model (Cox regression). Differences between methylation outcomes of patients were examined by use of generalised estimation equations for correlated and repeated measurement data. These multivariate and multilevel data of the primary tumour (when available) and multiple metastases were analysed with the SAS procedure GENMOD, assuming an exchangeable correlation structure (Diggle *et al*, 1994). Taking into account the explorative nature of this investigation, a significance level of 0.05 was used throughout. All statistical analyses were performed using SAS version 9.2 (Heidelberg, Germany).

## Results

One-hundred and twenty-two patients were included in this translational research project to evaluate *MGMT* promoter methylation. In this patient population, 63.1% were male and the mean age was 56 years (s.d. 12.8; range 23–80 years). Of these patients, 63.1% were treated with chemoimmune therapy, namely, TMZ plus interferon. Compared with the complete study population of the two DeCOG trials, there were no great differences between gender, age, or measures of clinical outcome such as RR or OS (Table 1) (Kaufmann *et al*, 2005; Spieth *et al*, 2008).

### MGMT promoter methylation did not differ significantly between primary tumours and metastases of the melanoma

Because little is yet known about possible differences between *MGMT* promoter methylation of different metastases or over the clinical course, we analysed the MGMT methylation status of primary tumours and subsequent metastases of a patient. In this evaluation, up to nine different metastases of a patient and different samples of each metastasis were used to investigate variation of methylation between primary and metastases. Altogether, 480 samples from 65 patients with at least two metastases were analysed. Of these, 209 samples were methylated and 271 unmethylated. Statistical analysis failed to reveal any significant difference between the *MGMT* methylation status of primary tumours and metastases or between different metastases of a patient (*P*=0.49 after adjustment for age and gender, Table 2). Hence, the *MGMT* methylation status seems not to change with clinical course, and for evaluation of the *MGMT* methylation status of the patient's melanoma the primary or a metastasis can be taken.

**Figure 2:**
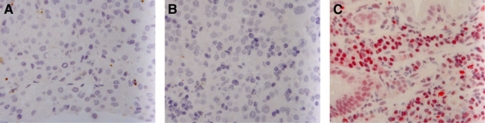
*O*^6^-methylguanine-DNA-methyltransferase expression by immunohistology in melanoma metastases with MGMT promotor methylation status analysed by COBRA. (**A**) COBRA analysis suggested unmethylated MGMT promotor; (**B**) or a methylated MGMT promotor. In comparision as positive staining control a colonic cancer biopsy was chosen (**C**). The immunohistochemical staining was carried out on paraffin-embedded tissue using a monoclonal anti-MGMT antibody and a Polymer-based Permanent AP Red Kit. Slides were shortly counterstained with Hämalaun.

In addition to *MGMT* promoter methylation, we examined several tissue samples for MGMT protein expression levels by immunohistochemistry. *O*^6^-methylguanine-DNA-methyltransferase expression was very low in all the melanoma samples tested, irrespective of the *MGMT* promoter methylation status determined or clinical outcome (Figure 2, Supplementary Table 2).

### MGMT promoter methylation did not correlate with clinical outcome

For statistical analysis of clinical response and patient survival, the first evaluable primary or metastasis of a patient was always included in the calculation to preserve the sample size. If, for methodological reasons, no valid methylation status was observed for that first sample the next patient sample was taken. Of 117 evaluable patients (clinical data were missing for five patients), six patients (5.1%) responded to treatment with a CR, 17 (14.5%) with a PR, 31 (26.5%) had SD, and 63 (53.9%) had progressive disease (PD) as best response to therapy.

Combined bisulphite restriction analysis evaluation of these samples revealed different grades of *MGMT* promoter methylation of the tumour DNA: 76 of 122 biopsy samples (62.3%) were unmethylated and 46 (37.7%) were methylated. The investigation included 27 primaries (22.1%) and 95 metastases. More information about patients and the clinical data are given in Table 3.

Correlation of the *MGMT* methylation status with clinical response to TMZ revealed no significant difference between therapy responders and non-responders in multivariate analysis adjusted for age and gender (*P*=0.26). Of 23 patients with either CR or PR as best response to therapy, 11 patients (47.8%) had a methylated MGMT promoter compared with 32 of 94 (34.0%) in the non-responder group (SD+PD). With regard to DCR, MGMT promoter methylation was observed for 42.6% of patients in group SD+PR+CR compared with 31.7% in the PD group (*P*=0.25) (Table 3).

**Figure 3:**
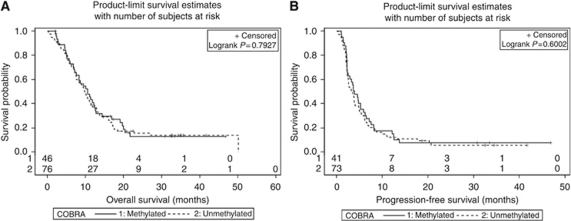
Kaplan–Meier curves for overall survival (OS, **A**) and progression-free survival (PFS, **B**) for the two groups of patients with methylated or unmethylated MGMT promoters, respectively, revealing no significant difference.

Kaplan–Meier curves revealed median OS of 10.5 months (95% CI: 7.2–12.5) for patients whose tumours were methylated at the MGMT promoter compared with a median of 9.7 months (95% CI: 7.6–11.4) for patients with an unmethylated MGMT promoter (Figure 3). There was, therefore, no significant difference between OS in these two groups (log-rank test: *P*=0.79). This finding was confirmed by multivariate analysis after adjustment for age and gender (Cox regression: *P*=0.31; HR 0.955 with 95% CI: 0.63–1.45). Similar results were obtained for PFS. Here, the Kaplan–Meier curves of the two patient groups were almost equal with a median time to progression of 3.4 months (95% CI: 2.4–4.0) for patients with an unmethylated promoter and 3.9 months (95% CI: 2.3–5.3) for patients with a methylated MGMT promoter (log-rank test: *P*=0.60) (Figure 3). Again this was confirmed by multivariate analysis after adjustment for age and gender (Cox regression: *P*=0.40; HR 0.91 with 95% CI: 0.61–1.36).

In univariate analysis we searched for other factors with possible effect on either methylation status or patient survival. Addition of interferon-*α* to TMZ treatment suggested a trend to better therapy response (*P*=0.10; OR 0.41; 95% CI: 0.14–1.2), but no effect for OS (*P*=0.77; HR 1.06; 95% CI: 0.7–1.6) or for PFS (*P*=0.53; HR 1.14; 95% CI: 0.76–1.7). This is in agreement with the clinical findings of the DeCOG trial, which revealed a better response for the combination with interferon that did not translate into a survival benefit (Kaufmann *et al*, 2005; Spieth *et al*, 2008).

In conclusion, the data reveal that *MGMT* promoter methylation does not correlate with clinical outcome of TMZ treatment in stage IV melanoma.

### MGMT promoter methylation correlated with tolerance of TMZ treatment

**Figure 4:**
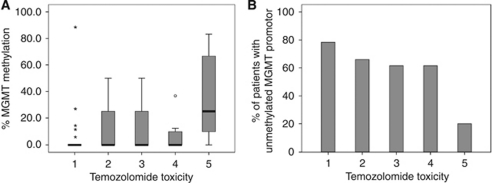
Correlation of MGMT methylation and TMZ tolerance. (**A**) MGMT methylation in the tolerance groups: 1=Excellent (no AE), 2=Good (AE but no dose reduction), 3=Satisfactory (dose reduction), 4=Moderate (therapy interrupted followed by dose reduction), and 5=Poor (therapy discontinued); (**B**) Percentage of patients with an unmethylated MGMT promoter in the five tolerance groups, demonstrating that MGMT methylation correlates with TMZ tolerance (*P*=0.007, OR 2.7 (95% CI: 1.32–5.7)).

Tolerance of therapy was documented in 111 of the 122 patients, and AEs were grouped into five categories including dose reduction, therapy interruption, and discontinuation (Table 4, Figure 4). Interestingly, we found an effect of MGMT promoter methylation status of the tumour biopsy on patients’ tolerance of TMZ. Patients with an unmethylated MGMT promoter developed fewer AEs than those with a methylated promoter. This was observed in univariate analysis (Table 3; *P*=0.045) and was confirmed by multivariate analysis which indicated that tolerance was associated with methylation status (*P*=0.007; OR 2.7 with 95% CI: 1.32–5.7) and gender (*P*=0.001; OR 3.4 with 95% CI: 1.63–7.11) but not with age (*P*=0.86). An unmethylated MGMT promoter was shown to be associated with, on average, 2.7-fold better tolerance of therapy (Figure 4).

## Discussion

Gene promoter methylation of the DNA repair enzyme MGMT correlates with clinical response to TMZ treatment in combination with radiotherapy and significantly alters OS of patients with glioblastoma (Hegi *et al*, 2005). In melanoma, results are still controversial. Using a biochemical method to measure MGMT activity, Middleton *et al* (1998) found no correlation with clinical response. In contrast, Ma *et al* (2003) reported immunohistochemical staining with a monoclonal antibody and described an association of MGMT expression level with clinical response to DTIC chemotherapy (*P*=0.05), noting that MGMT nuclear staining was difficult to evaluate. Rietschel *et al* (2008) also investigated MGMT expression by a methylation-dependent method and by immunohistochemistry and found no association with response in a limited number of patients. Here, we investigated MGMT gene silencing by methylation of its promoter using the COBRA technique for 122 metastatic melanoma patients with first-line TMZ treatment in two large multicenter trials. No association between MGMT promoter methylation and clinical response was detectable. In addition, neither PFS nor OS differed between patients with a methylated or unmethylated MGMT promoter region in the melanoma biopsy. Immunohistochemical analysis of MGMT expression in a series of melanoma biopsies using a specific monoclonal antibody revealed generally low MGMT expression, irrespective of MGMT promoter methylation status. In accordance with this observation, melanoma is known to belong to tumours with the lowest average MGMT expression (in contrast, for example, with colon and breast carcinoma) (Kaina *et al*, 2007). Previous *in vitro* studies on melanoma cell lines could find expression of MGMT (Mhaidat *et al*, 2007). However, cell lines might have changed their expression patterns with culturing. It is therefore conceivable that low MGMT expression levels in melanoma also reflect minor importance of MGMT for TMZ resistance in this disease. In line with this concept is the observation that addition of the MGMT inhibitor *O*^6^-bromothenylguanine (lomeguatrib) to TMZ in a phase II trial did not improve RR or PFS (Ranson *et al*, 2007). In melanoma cell lines, TMZ has been shown to induce cell cycle arrest associated with accumulation of p53 and p21, but which is not followed by apoptosis (Mhaidat *et al*, 2007). Chemoresistance to TMZ could, therefore, also be based on other resistance mechanisms, for example, the known apoptosis resistance of melanomas in general, and not so much be modulated by the low MGMT activity. In addition, TMZ-induced *O*^6^-methylation causes double-strand breaks, which could be repaired by other DNA repair enzymes such as the homologous repair (HR) enzymes XRCC2 and BRCA2. Roos *et al* (2009) could show that HR-mutated glioma cells are hypersensitive to *O*^6^-methylguanin triggered cell death induced by TMZ. Therefore, HR can be considered as a mechanism that causes tolerance of *O*^6^-methylguanin adducts. BRCA2 mutations are not very common in melanoma, but among carriers of the N991D change of the BRCA2 gene the risk to develop melanoma is increased (Debniak *et al*, 2008). However, whether these patients were sensitive to TMZ is not reported.

The MGMT methylation status of the tumour could therefore be more a patient phenotype having little to do with MGMT expression level and TMZ chemoresistance. In agreement with this interpretation, we found no significant differences between the MGMT methylation status of melanoma primaries or metastases in 65 patients with up to 9 metastases. This confirms the findings of Rastetter *et al* (2007) but is in contrast with those of Kohonen-Corish *et al* (2006) who described heterogeneity in MGMT gene methylation between melanoma metastases from the same patient for a limited number of 11 patients.

*O*^6^-methylguanine-DNA-methyltransferase methylation status as a host-specific rather than tumour-specific condition explains why MGMT promoter methylation of the tumour was highly associated with TMZ tolerance in patients requiring more dose reductions or discontinuations. *O*^6^-methylguanine-DNA-methyltransferase expression in peripheral blood mononuclear cells (PBMCs) is a good surrogate marker for that in blood progenitor cells (Gerson *et al*, 1985) and could explain TMZ haematologic toxicity. Combination of alkylating agents with MGMT inhibitors, for example, the pseudosubstrates *O*^6^-benzylguanine and lomeguatrib, led to increased occurrence of AEs and dose reductions in clinical trials. The effort to improve chemosensitivity towards alkylating agents has therefore been paralleled by increased toxicity (Ranson *et al*, 2007; Tawbi and Kirkwood, 2007). Toxicity is in contrast with response of melanoma correlated with MGMT activity.

In glioblastoma patients, the correlation between DNA methylation in tumour tissue and free circulating DNA of sera was highly significant (Balana *et al*, 2003; Ramirez *et al*, 2003). Hence, besides evaluation of MGMT methylation in PBMCs, future investigations should test whether the toxicity of TMZ could possibly be predicted by measuring the MGMT methylation status of free DNA in the serum. This could lead to an easy tool for prediction of a patient's tolerance of planned chemotherapy with TMZ and lead to preselection of patients to be treated.

## Statement of translational relevance

This work demonstrates for the first time that, in melanoma, silencing of the *MGMT* promoter correlates with TMZ toxicity. This could mean that *MGMT* methylation of the tumour is host-specific rather than tumour-specific. In agreement with this we found no difference between *MGMT* methylation status in primaries and different metastases of a patient. In contrast to glioblastoma, we found that *MGMT* methylation status was not important in TMZ response or survival of patients.

Because DTIC and TMZ are still the standard therapy for melanoma, however, determination of *MGMT* methylation could indicate for which patients these treatments should not be given, because of expected toxicity. This could possibly be measured in patients’ blood and should be confirmed in a prospective trial.

## Figures and Tables

**Table 1:** Comparison of the study populations in the two DeCOG trials with the patients in this translational research project

**Study**	**Number of patients**	**Gender male (%)**	**Age median (years)**	**Response rate (%)**	**Overall survival (months)**
DeCOG I (2 arms: TMZ/TMZ+IFN) (Kaufmann *et al*, 2005)	294	64.0/59.4 *P*=0.45	56.0/54.5 *P*=0.86	13.4/24.1 *P*=0.036	8.4/9.7 *P*=0.16
DeCOG II (TMZ+pegIFN) (Spieth *et al*, 2008)	124	66.7	55.5	18.1	9.4
Translational research project	122	63.1	56	19.6	9.7/10.5 (meth/unmeth MGMT)

Abbreviations: DeCOG=Dermatologic Cooperative Oncology Group; MGMT=*O*^6^-methylguanine-DNA-methyltransferase; TMZ=temozolomide; IFN=interferon-*α*; pegIFN=pegylated interferon-*α*; meth/unmeth=methylated/unmethylated.

**Table 2:** Results from determination of *MGMT* methylation of primaries and metastases

**Groups**	**All tumour samples (*n*)**	**Primaries (*n*, %)**	**Metastases (*n*, %)**	**Mean difference (95% CI)**	**Level of significance between groups**
All	480	59	421		*P*=0.491^a^
Methylated	209	24	185	−0.66	
Unmethylated	271	35	236	(−2.55/1.22)	

Abbreviations: CI=confidence interval; MGMT=*O*^6^-methylguanine-DNA-methyltransferase.

a After adjustment for age and gender.

**Table 3:** Description of patient population with methylated and unmethylated *MGMT* promoters

**Groups**	**Mean age (range)**	**Gender**	**Treatment**	**RR^a^ (*n*=117)**	**DCR^a^ (*n*=117)**
All (*n*=122)	56 years (23–80)	63.1% male (*n*=77)	63.1% TMZ+INF (*n*=77)	CR+PR: 19.7% (*n*=23), SD+PD: 80.3% (*n*=94)	CR+PR+SD: 46.2% (*n*=54), PD: 53.8% (*n*=63)
Methylated (*n*=46, 37.7%)	58.1 years (23–80)	58.7% male (*n*=27)	67.4% (*n*=31)	CR+PR: 25.6% (*n*=11), SD+PD: 74.4% (*n*=32)	CR+PR+SD: 53.5% (*n*=23), PD: 46.5% (*n*=20)
Unmethylated (*n*=76, 62.3%)	54.7 years (30–78)	65.8% male (*n*=50)	60.5% (*n*=46)	CR+PR: 16.2% (*n*=12), SD+PD: 83.8% (*n*=62)	CR+PR+SD: 41.9% (*n*=31), PD: 58.1% (*n*=43)
Level of significance of differences between groups	*P*=0.29	*P*=0.52	*P*=0.56	*P*=0.26	*P*=0.25

Abbreviations: TMZ=temozolomide; INF=interferon-*α*; RR=response rate; DCR=disease control rate; CR=complete response; PR=partial response; SD=stable disease; PD=progressive disease; MGMT=*O*^6^-methylguanine-DNA-methyltransferase.

a Five patients with missing clinical data at best response to therapy.

**Table 4:** Tolerance of TMZ, and *MGMT* methylation status

**Groups**	**Excellent**	**Good**	**Satisfactory**	**Moderate**	**Poor**	***χ*^2^ (Fisher's exact)**
Methylated (*n*=43), *n* (%)	4 (9.3)	18 (41.9)	9 (21.0)	7 (16.3)	5 (11.6)	*P*=0.045
Unmethylated (*n*=68), *n* (%)	17 (25.0)	30 (44.1)	14 (20.6)	6 (8.8)	1 (1.5)	

Abbreviations: MGMT=*O*^6^-methylguanine-DNA-methyltransferase; TMZ=temozolomide.

Tolerance during TMZ treatment of patients with a methylated or unmethylated MGMT promoter. Tolerance groups were defined by the grade of adverse event, which resulted in treatment modifications: *Excellent*=no adverse events, *Good*=adverse event, no dose reduction required, *Satisfactory*=dose reduction required, *Moderate*=therapy interrupted, followed by subsequent dose reduction, and *Poor*=therapy discontinued. Patients with unmethylated MGMT promoter tolerated therapy significantly better (*P*=0.045) (Figure 3).
