# Supplementary material for: MGMT gene promoter methylation correlates with tolerance of temozolomide treatment in melanoma but not with clinical outcome
Source: Br J Cancer. 2010 Aug 24;103(6):820–6. doi: 10.1038/sj.bjc.6605796 (PMC2966614; doi:10.1038/sj.bjc.6605796)
Supplement: Supplementary Table 1 [file 6605796x1.doc]

# Supplemental Table 1: Results from bisulfite sequencing of five methylated and five unmethylated samples in COBRA analysis for quality control

| **Sample** | Clone 1 | 2 | 3 | 4 | 5 | 6 | 7 | 8 | 9 | 10 | On average |
| --- | --- | --- | --- | --- | --- | --- | --- | --- | --- | --- | --- |
| 39 | 0 | 0 | 1 | 1 | 10 | 4 | 0 | 0 | 9 | 9 | 3.4 |
| 713 | 12 | 2 | 11 | 2 | 2 | 11 | 11 | 12 | 12 | 2 | 7.7 |
| 433 | 0 | 11 | 0 | 1 | 1 | 1 | 11 | 3 | 11 | 12 | 5.1 |
| 531 | 2 | 1 | 0 | 1 | 1 | 12 | 1 | 13 | 0 | 0 | 3.1 |
| 137 | 0 | 0 | 11 | 0 | 11 | 11 | 11 | 0 | 0 | 0 | 4.4 |
| 681 | 11 | 0 | 12 | 2 | 2 | 0 | 11 | 0 | 0 | 0 | 3.8 |
| 150 | 11 | 11 | 11 | 11 | 11 | 11 | 11 | 11 | 11 | 14 | 11.3 |
| 648 | 12 | 12 | 12 | 11 | 12 | 11 | 12 | 12 | 12 | 12 | 11.8 |
| 401 | 11 | 2 | 11 | 0 | 12 | 13 | 14 | 12 | 12 | 12 | 9.9 |
| 395 | 11 | 2 | 11 | 12 | 12 | 12 | 4 | 12 | 11 | 12 | 9.9 |

For quality control five samples each, defined by COBRA as unmethylated and methylated, respectively, were bisulfite sequenced. The COBRA PCR products were extracted from the gel and cloned. The first five samples (samples 39–137) were classified as “methylated” by COBRA, the latter five (samples 681–395) as “unmethylated”. Four of the five “methylated” classified COBRA samples revealed a methylation degree of ≥50% in COBRA analysis and 9.9–11.8 methylated CpGs in bisulfite sequencing. The fifth sample revealed only 10% methylation with 3.8 methylated CpGs. The five samples which were classified as unmethylated by COBRA analysis showed methylation between 3.1 and 7.7 methylated CpGs of 33 CpGs analyzed (Fig.1b). Together, these results confirmed that the COBRA assay adequately represents the genomic methylation level of the *MGMT* promoter.
