# Supplementary material for: MGMT gene promoter methylation correlates with tolerance of temozolomide treatment in melanoma but not with clinical outcome
Source: Br J Cancer. 2010 Aug 24;103(6):820–6. doi: 10.1038/sj.bjc.6605796 (PMC2966614; doi:10.1038/sj.bjc.6605796)
Supplement: Supplementary Table 2 [file 6605796x2.doc]

# Supplemental Table 2: Results of immunohistochemistry staingings of MGMT and patient clinical data

| **Sample** | **COBRA result** | **IHC staining** | **Survival (months)** |
| --- | --- | --- | --- |
| 1 | unmethylated | negative | at least 21* |
| 2 | unmethylated | negative | 10 |
| 3 | unmethylated | negative | 7 |
| 4 | unmethylated | negative | 3 |
| 5 | methylated | negative | at least 47 |
| 6 | unmethylated | negative | 18 |
| 7 | unmethylated | negative | at least 35 |
| 8 | methylated | negative | at least 34 |

* at last contact still alive

IHC stainings of all tested samples were negative – independent of MGMT promoter methylation and survival under temozolomide treatment.
